# Mol­ecular and crystal structure, optical properties and DFT studies of 1,4-dimeth­oxy-2,5-bis­[2-(4-nitro­phen­yl)ethen­yl]benzene

**DOI:** 10.1107/S205698902000674X

**Published:** 2020-05-29

**Authors:** Georgii Bogdanov, Evgenii Oskolkov, Jenna Bustos, Viktor Glebov, John P. Tillotson, Tatiana V. Timofeeva

**Affiliations:** a Department of Chemistry, New Mexico Highlands University, Las Vegas, New Mexico, 87701, USA; b Department of Chemical and Biomolecular Engineering, University of California Irvine, Irvine, California, 92617, USA; c School of Chemistry and Biochemistry, Georgia Institute of Technology, Atlanta, Georgia, 30332, USA

**Keywords:** crystal structure, di­meth­oxy­benzene, two-photon absorption, Hirshfeld surface, DFT calculations, absorption and emission spectra

## Abstract

The title compound DBNB, C_24_H_20_N_2_O_6_, has been crystallized and studied by X-ray diffraction, spectroscopic and computational methods. In the title mol­ecule, which is based on a 1,4-distyryl-2,5-di­meth­oxy­benzene core with *p*-nitro-substituted terminal benzene rings, the dihedral angle between mean planes of the central fragment and the terminal phenyl ring is 16.46 (6)°. The crystal packing is stabilized by π–π inter­actions. DFT calculations at the B3LYP/6–311 G(d,p) level of theory were used to compare the optimized structures with the experimental data. Energy parameters, including HOMO and LUMO energies, their difference, and vertical excitation and emission energies were obtained.

## Abstract 2

In the title mol­ecule, which is based on a 1,4-distyryl-2,5-di­meth­oxy­benzene core with *p*-nitro-substituted terminal benzene rings, the dihedral angle between mean planes of the central fragment and the terminal phenyl ring is 16.46 (6)°. The crystal packing is stabilized by π–π inter­actions.

## Chemical context   

One method for the design of the organic two-photon absorbing (TPA) mol­ecules is Donor–π-Bridge–Acceptor–π-Bridge–Donor or Acceptor–π-bridge–Donor–π-bridge–Acceptor (He *et al.*, 2008 ▸). Specific spectroscopic properties of such mol­ecules make them useful for applications in different areas. For instance, about half a century ago it was found that the title compound and other substituted distyryl­benzenes would be highly efficient wavelength shifters in organic liquid scintillators (Nakaya *et al.*, 1966 ▸). It is important to mention that some mol­ecules with such general structure possess not only plasminogen activator (tPA) activity but also demonstrate light emission, which make them useful for organic light-emitting diodes (OLEDs) (Cárdenas *et al.*, 2019 ▸) and/or chemical sensors (Xu *et al.*, 2013 ▸). For instance, for a mol­ecule similar to the title mol­ecule, 1,4-dimeth­oxy-2,5-bis­(4′-di­chloro­styr­yl)benzene, blue fluorescence emission was found, which makes it a prospective candidate for cell imaging. Another phenyl­eneethenylene derivative, 2,5-dimeth­oxy-1,4-bis­[2-(4-carboxyl­atestyr­yl)]benzene, for which two polymorphs and one DMF solvate have been studied, demonstrated three different types of emission, depending on the mol­ecular packing in the crystal (Cárdenas *et al.*, 2019 ▸). On this basis, we considered that an investigation of the mol­ecular structure and crystal packing of the title compound would be useful for correlating its structural characteristics to its spectroscopic properties.
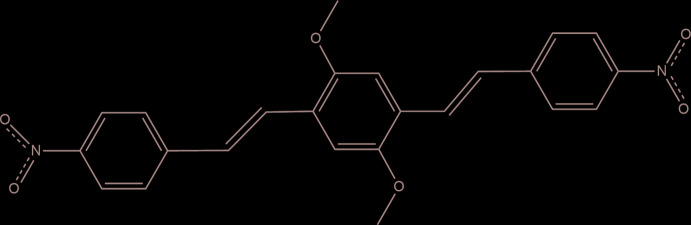


## Structural commentary   

**Figure 1:**
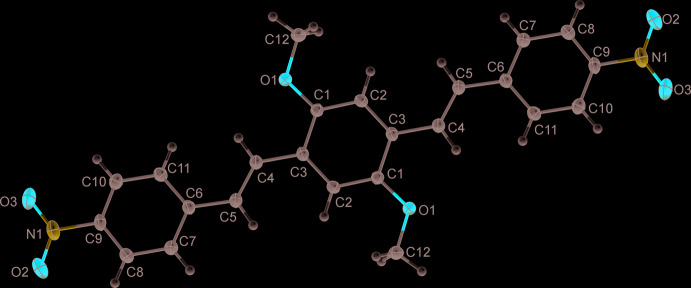
A view of the mol­ecular structure of the title compound with the atom-labeling scheme. Displacement ellipsoids are drawn at the 50% probability level.

The mol­ecular structure of DBDB is presented in Fig. 1 ▸. The mol­ecule lies on an inversion center and shows a slight deviation from planarity. The dihedral angle formed by mean planes of the central fragment and the terminal benzene ring is 16.46 (6)°. The meth­oxy group is rotated by 3.77 (11)° and the nitro group by 15.99 (8)° with respect to the central ring and the terminal benzene ring, respectively. In a similar compound with *para*-chlorine substitution, the angles between the central and terminal aromatic rings are 43.82 and 67.38° (Xu *et al.*, 2013 ▸), whereas in closely related structures these angles vary from 11.97 to 35.75° (Cárdenas *et al.*, 2019 ▸), demonstrating the flexibility of this type of mol­ecule, even in the solid state.

## Supra­molecular features   

**Figure 2:**
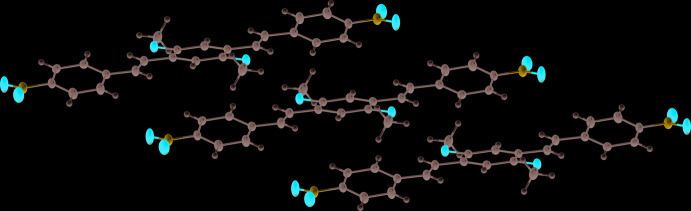
Ladder-like stack of DBDB mol­ecules; the distance between the mean planes of the central phenyl rings within the stack is 3.451 (1) Å.

**Figure 3:**
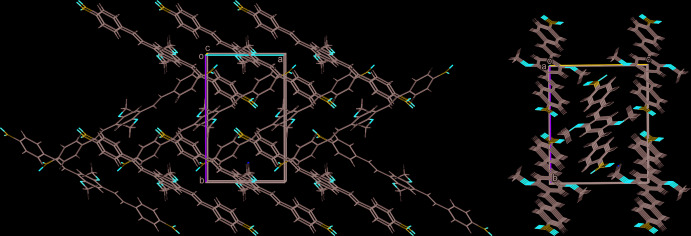
The packing in the crystal of the title compound.

In the crystal, the DBDB mol­ecules are packed into ladder-like stacks (Fig. 2 ▸) along the *a*-axis direction, which in turn build a parquet-like structure (Fig. 3 ▸). An inter­molecular distance of 3.451 (1) Å is found between the mean planes of the central rings in the mol­ecular stacks, with a separation between the centroids of the central ring and the terminal benzene ring of 3.899 (1) Å, which suggests the presence of π–π inter­actions between the mol­ecules.

## Database survey   

A search of the Cambridge Crystallographic Database (CSD version 5.40, update of September 2019; Groom *et al.*, 2016 ▸) for the title mol­ecule returned no results. Two entries for compounds with the same core and unsubstituted terminal rings were found. Over 30 entries were found for variously substituted mol­ecules with the same core, of which 10 entries correspond to *para*-substituted terminal aromatic groups. Among them halogen-substituted mol­ecules [refcodes: VIQCAB (Xu *et al.*, 2013 ▸), ODOHOG (Sun *et al.*, 2013 ▸), ODOJAU (Sun *et al.*, 2013 ▸)], as well as mol­ecules with cyano (OBUHAV; Xu *et al.*, 2013 ▸), carboxyl (TOJDEE, TOJDII; Cárdenas *et al.*, 2019 ▸) and alkyl­carboxyl­ate (TOJCUT; Cárdenas *et al.*, 2019 ▸) groups in the *para*-position have been reported. Most of the mol­ecules demonstrate dihedral angles between the central fragment and the terminal rings ranging from 5.0 (1) to 36.1 (1)°. One notable exception is the chlorine-substituted compound (VIQCAB; Xu *et al.*, 2013 ▸), for which the angles between central and the terminal aromatic rings are 43.82 (16) and 67.38 (17)°.

## Optical studies in solution   

**Figure 4:**
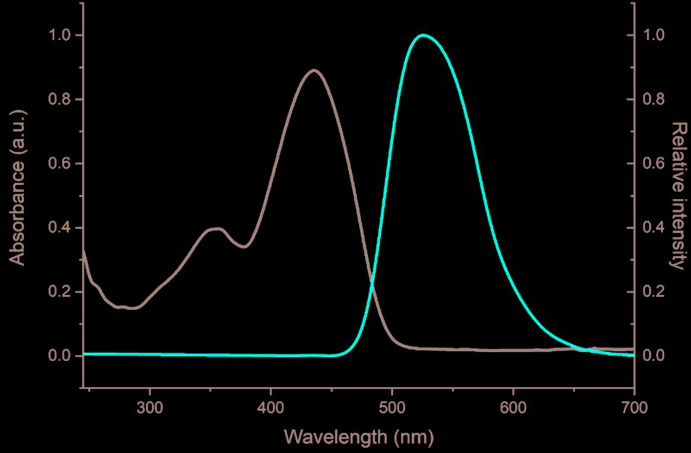
Normalized absorption (black) and emission (red) spectra of the title compound measured in dioxane solution.

A solution of the title compound in dioxane (at 10 m*M* concentration) in a quartz sample cuvette (10 mm optical path length) was used for optical absorption and emission studies. All measurements were carried out at ambient temperature. The corresponding spectra are shown in Fig. 4 ▸. Peak positions, as well as band shapes are in good agreement with those previously reported (Nakaya *et al.*, 1966 ▸). Fluorescence was measured at the excitation wavelength of 434 nm, chosen from the absorption spectrum, and had a maximum at 525 nm. The E_0–0_ transition energy was estimated to be at 483 nm (2.57 eV).

## DFT calculations   

**Figure 5:**
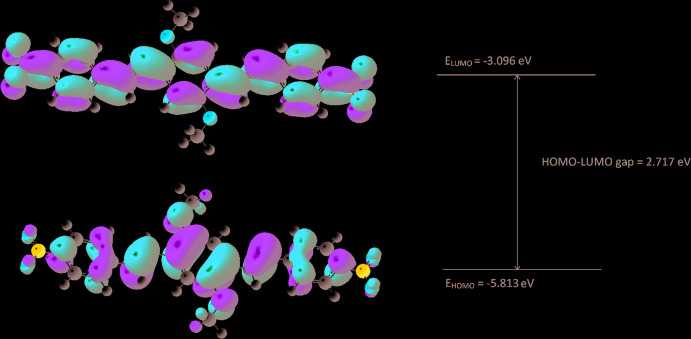
HOMO and LUMO orbitals with corresponding energy values and gap.

In an effort to further elucidate the nature of the electronic radiative transitions in the title compound, DFT and time-dependent (TD) DFT calculations were carried out with *GAUSSIAN 16* software (Frisch *et al.*, 2016 ▸). The standard B3LYP functional with the 6-311G(d,p) basis set was used to optimize both the ground and first excited states of the title mol­ecule and to obtain vertical excitation and emission energies, HOMO (*E*_HOMO_) and LUMO (*E*_LUMO_) energies and their difference (Fig. 5 ▸). All of the calculated parameters are for the gas phase of the title compound. Both optimized geometries were confirmed to be the true minima *via* vibrational frequency analysis. The summary of calculated energy parameters is presented in Table 1 ▸. The calculated geometry parameters (bond lengths and angles) are in good agreement with the experimental data (Table 2 ▸).

## Synthesis and crystallization   

The synthesis of title compound was carried out as described in the literature (Nakaya *et al.*, 1966 ▸; Caruso *et al.*, 2005 ▸). The obtained material was recrystallized by slow evaporation of ethanol solution giving dark-red block-shaped crystals.

## Refinement   

Crystal data, data collection and structure refinement details are summarized in Table 3 ▸. H atoms were placed in calculated positions (0.95–0.98 Å) and refined as riding with *U*_iso_(H) = 1.2*U*_eq_(C) or 1.5*U*_eq_(C-meth­yl).

## Supplementary Material

Crystal structure: contains datablock(s) I. DOI: 10.1107/S205698902000674X/yk2130sup1.cif

Structure factors: contains datablock(s) I. DOI: 10.1107/S205698902000674X/yk2130Isup2.hkl

Supporting information file. DOI: 10.1107/S205698902000674X/yk2130Isup3.cml

CCDC reference: 2004742

Additional supporting information:  crystallographic information; 3D view; checkCIF report

## Figures and Tables

**Table 1:** Selected energy parameters (gas phase)

Total Energy (eV)	−40479.535
*E*_HOMO_ (eV)	−5.813
*E*_LUMO_ (eV)	−3.096
HOMO–LUMO gap (eV)	2.717
S_0_–S_1_ vertical excitation (nm)	497.44
S_1_–S_0_ vertical emission (nm)	546.03

**Table 2:** Selected X-ray and DFT ground-state geometry parameter (Å, °) comparison

Bonds/angles	Experimental	Calculated
O1—C1	1.3663 (14)	1.3652
O1—C12	1.4248 (15)	1.4207
O2—N1	1.2321 (16)	1.2253
N1—C9	1.4665 (16)	1.4723
		
C5—C4—C3	127.07 (11)	126.76
C4—C5—C6	125.13 (11)	126.38
C1—O1—C12	117.68 (9)	119.05
C8—C9—N1	118.92 (11)	119.26

**Table 3:** Experimental details

Crystal data	
Chemical formula	C_24_H_20_N_2_O_6_
*M*_r_	432.42
Crystal system, space group	Monoclinic, *P*2_1_/*n*
Temperature (K)	100
*a*, *b*, *c* (Å)	7.9074 (10), 12.4794 (16), 10.6248 (14)
β (°)	102.394 (3)
*V* (Å^3^)	1024.0 (2)
*Z*	2
Radiation type	Mo *K*α
μ (mm^−1^)	0.10
Crystal size (mm)	0.22 × 0.15 × 0.11

Data collection	
Diffractometer	Bruker APEXII CCD
Absorption correction	Multi-scan (*SADABS*; Bruker, 2016 ▸)
*T*_min_, *T*_max_	0.653, 0.746
No. of measured, independent and observed [*I* > 2σ(*I*)] reflections	32090, 3460, 2542
*R*_int_	0.055
(sin θ/λ)_max_ (Å^−1^)	0.738

Refinement	
*R*[*F* ^2^ > 2σ(*F* ^2^)], *wR*(*F* ^2^), *S*	0.048, 0.145, 1.04
No. of reflections	3460
No. of parameters	146
H-atom treatment	H-atom parameters constrained
Δρ_max_, Δρ_min_ (e Å^−3^)	0.49, −0.21

Computer programs: *APEX3* (Bruker, 2016 ▸), *SAINT* (Bruker, 2016 ▸), *SHELXT2017/1* (Sheldrick, 2015*a* ▸), *SHELXL2017/1* (Sheldrick, 2015*b* ▸), *OLEX2* (Dolomanov *et al.*, 2009 ▸), *Mercury* (Macrae *et al.*, 2020 ▸).

## supplementary crystallographic information

### Crystal data

**Table:**

C_24_H_20_N_2_O_6_	*F*(000) = 452
*M**_r_* = 432.42	*D*_x_ = 1.403 Mg m^−^^3^
Monoclinic, *P*2_1_/*n*	Mo *K*α radiation, λ = 0.71073 Å
*a* = 7.9074 (10) Å	Cell parameters from 6481 reflections
*b* = 12.4794 (16) Å	θ = 2.6–31.3°
*c* = 10.6248 (14) Å	µ = 0.10 mm^−^^1^
β = 102.394 (3)°	*T* = 100 K
*V* = 1024.0 (2) Å^3^	Block, red
*Z* = 2	0.22 × 0.15 × 0.11 mm

### Data collection

**Table:**

Bruker APEXII CCD diffractometer	2542 reflections with *I* > 2σ(*I*)
φ and ω scans	*R*_int_ = 0.055
Absorption correction: multi-scan (SADABS; Bruker, 2016)	θ_max_ = 31.7°, θ_min_ = 2.6°
*T*_min_ = 0.653, *T*_max_ = 0.746	*h* = −11→11
32090 measured reflections	*k* = −18→18
3460 independent reflections	*l* = −15→15

### Refinement

**Table:**

Refinement on *F*^2^	Primary atom site location: dual
Least-squares matrix: full	Secondary atom site location: difference Fourier map
*R*[*F*^2^ > 2σ(*F*^2^)] = 0.048	Hydrogen site location: inferred from neighbouring sites
*wR*(*F*^2^) = 0.145	H-atom parameters constrained
*S* = 1.03	*w* = 1/[σ^2^(*F*_o_^2^) + (0.0723*P*)^2^ + 0.3749*P*] where *P* = (*F*_o_^2^ + 2*F*_c_^2^)/3
3460 reflections	(Δ/σ)_max_ < 0.001
146 parameters	Δρ_max_ = 0.49 e Å^−^^3^
0 restraints	Δρ_min_ = −0.21 e Å^−^^3^

### Special details

**Table:**

Geometry. All esds (except the esd in the dihedral angle between two l.s. planes) are estimated using the full covariance matrix. The cell esds are taken into account individually in the estimation of esds in distances, angles and torsion angles; correlations between esds in cell parameters are only used when they are defined by crystal symmetry. An approximate (isotropic) treatment of cell esds is used for estimating esds involving l.s. planes.

### Fractional atomic coordinates and isotropic or equivalent isotropic displacement parameters (Å^2^)

**Table:**

	*x*	*y*	*z*	*U*_iso_*/*U*_eq_	
O1	1.12432 (11)	0.50902 (7)	0.27624 (8)	0.0223 (2)	
O2	−0.13219 (13)	0.91355 (10)	0.42105 (12)	0.0423 (3)	
O3	−0.08612 (15)	0.85312 (11)	0.61675 (12)	0.0453 (3)	
N1	−0.04597 (14)	0.86252 (10)	0.51181 (12)	0.0301 (3)	
C1	1.05803 (14)	0.50680 (9)	0.38485 (11)	0.0176 (2)	
C2	0.90285 (14)	0.55533 (9)	0.39523 (11)	0.0185 (2)	
H2	0.836944	0.592801	0.323366	0.022*	
C3	0.84235 (14)	0.54988 (9)	0.50948 (11)	0.0178 (2)	
C10	0.18811 (15)	0.73214 (10)	0.58176 (12)	0.0215 (2)	
H10	0.137655	0.713154	0.652137	0.026*	
C4	0.67887 (14)	0.59782 (10)	0.52387 (11)	0.0202 (2)	
H4	0.640701	0.580352	0.600263	0.024*	
C6	0.41503 (15)	0.71106 (10)	0.46101 (11)	0.0196 (2)	
C11	0.33818 (15)	0.68241 (9)	0.56370 (11)	0.0198 (2)	
H11	0.389852	0.627975	0.621909	0.024*	
C9	0.11358 (14)	0.81049 (10)	0.49411 (12)	0.0217 (2)	
C5	0.57748 (16)	0.66340 (10)	0.44141 (12)	0.0225 (2)	
H5	0.613011	0.680685	0.363923	0.027*	
C8	0.18426 (16)	0.84114 (11)	0.39171 (12)	0.0249 (3)	
H8	0.131401	0.895451	0.333775	0.030*	
C7	0.33514 (17)	0.79047 (11)	0.37539 (12)	0.0252 (3)	
H7	0.384749	0.810152	0.304835	0.030*	
C12	1.03435 (16)	0.57061 (12)	0.16982 (12)	0.0269 (3)	
H12A	1.097995	0.568337	0.100234	0.040*	
H12B	1.025026	0.645017	0.197031	0.040*	
H12C	0.918153	0.540834	0.138992	0.040*	

### Atomic displacement parameters (Å^2^)

**Table:**

	*U*^11^	*U*^22^	*U*^33^	*U*^12^	*U*^13^	*U*^23^
O1	0.0212 (4)	0.0287 (5)	0.0177 (4)	0.0069 (3)	0.0058 (3)	0.0033 (3)
O2	0.0253 (5)	0.0519 (7)	0.0485 (7)	0.0170 (5)	0.0054 (5)	0.0069 (5)
O3	0.0363 (6)	0.0580 (8)	0.0491 (7)	0.0163 (5)	0.0260 (5)	0.0067 (6)
N1	0.0192 (5)	0.0331 (6)	0.0387 (6)	0.0057 (4)	0.0082 (4)	−0.0013 (5)
C1	0.0175 (5)	0.0183 (5)	0.0172 (5)	0.0007 (4)	0.0040 (4)	−0.0014 (4)
C2	0.0168 (5)	0.0200 (5)	0.0180 (5)	0.0027 (4)	0.0018 (4)	0.0010 (4)
C3	0.0152 (5)	0.0181 (5)	0.0198 (5)	0.0018 (4)	0.0029 (4)	−0.0014 (4)
C10	0.0191 (5)	0.0228 (6)	0.0237 (5)	−0.0025 (4)	0.0071 (4)	−0.0014 (4)
C4	0.0180 (5)	0.0234 (5)	0.0195 (5)	0.0026 (4)	0.0049 (4)	−0.0007 (4)
C6	0.0167 (5)	0.0211 (5)	0.0206 (5)	0.0023 (4)	0.0033 (4)	−0.0012 (4)
C11	0.0180 (5)	0.0199 (5)	0.0212 (5)	0.0000 (4)	0.0034 (4)	0.0003 (4)
C9	0.0148 (5)	0.0246 (6)	0.0257 (6)	0.0028 (4)	0.0045 (4)	−0.0043 (4)
C5	0.0211 (5)	0.0250 (6)	0.0235 (6)	0.0059 (4)	0.0092 (4)	0.0036 (4)
C8	0.0227 (6)	0.0284 (6)	0.0235 (6)	0.0083 (5)	0.0044 (4)	0.0030 (5)
C7	0.0247 (6)	0.0301 (6)	0.0225 (6)	0.0085 (5)	0.0091 (5)	0.0052 (5)
C12	0.0212 (5)	0.0388 (7)	0.0211 (6)	0.0048 (5)	0.0055 (4)	0.0086 (5)

### Geometric parameters (Å, º)

**Table:**

O1—C1	1.3663 (14)	C4—C5	1.3337 (16)
O1—C12	1.4248 (15)	C6—C11	1.4043 (16)
O2—N1	1.2321 (16)	C6—C5	1.4704 (16)
O3—N1	1.2287 (16)	C6—C7	1.4007 (17)
N1—C9	1.4665 (16)	C11—H11	0.9500
C1—C2	1.3938 (15)	C9—C8	1.3796 (18)
C1—C3^i^	1.4148 (16)	C5—H5	0.9500
C2—H2	0.9500	C8—H8	0.9500
C2—C3	1.3986 (16)	C8—C7	1.3936 (17)
C3—C4	1.4619 (15)	C7—H7	0.9500
C10—H10	0.9500	C12—H12A	0.9800
C10—C11	1.3885 (16)	C12—H12B	0.9800
C10—C9	1.3910 (17)	C12—H12C	0.9800
C4—H4	0.9500		
			
C1—O1—C12	117.68 (9)	C10—C11—C6	121.25 (11)
O2—N1—C9	118.37 (12)	C10—C11—H11	119.4
O3—N1—O2	123.52 (12)	C6—C11—H11	119.4
O3—N1—C9	118.11 (11)	C10—C9—N1	118.55 (11)
O1—C1—C2	124.19 (10)	C8—C9—N1	118.92 (11)
O1—C1—C3^i^	115.54 (10)	C8—C9—C10	122.53 (11)
C2—C1—C3^i^	120.27 (10)	C4—C5—C6	125.13 (11)
C1—C2—H2	119.4	C4—C5—H5	117.4
C1—C2—C3	121.23 (10)	C6—C5—H5	117.4
C3—C2—H2	119.4	C9—C8—H8	120.8
C1^i^—C3—C4	118.41 (10)	C9—C8—C7	118.35 (11)
C2—C3—C1^i^	118.50 (10)	C7—C8—H8	120.8
C2—C3—C4	123.08 (10)	C6—C7—H7	119.4
C11—C10—H10	120.9	C8—C7—C6	121.27 (11)
C11—C10—C9	118.26 (11)	C8—C7—H7	119.4
C9—C10—H10	120.9	O1—C12—H12A	109.5
C3—C4—H4	116.5	O1—C12—H12B	109.5
C5—C4—C3	127.07 (11)	O1—C12—H12C	109.5
C5—C4—H4	116.5	H12A—C12—H12B	109.5
C11—C6—C5	122.95 (11)	H12A—C12—H12C	109.5
C7—C6—C11	118.34 (10)	H12B—C12—H12C	109.5
C7—C6—C5	118.69 (11)		
			
O1—C1—C2—C3	179.95 (11)	C11—C10—C9—N1	179.35 (11)
O2—N1—C9—C10	−164.27 (12)	C11—C10—C9—C8	−0.84 (19)
O2—N1—C9—C8	15.92 (19)	C11—C6—C5—C4	−7.6 (2)
O3—N1—C9—C10	15.98 (19)	C11—C6—C7—C8	0.74 (19)
O3—N1—C9—C8	−163.83 (13)	C9—C10—C11—C6	0.93 (18)
N1—C9—C8—C7	−179.49 (12)	C9—C8—C7—C6	−0.6 (2)
C1—C2—C3—C1^i^	−0.34 (18)	C5—C6—C11—C10	177.54 (11)
C1—C2—C3—C4	−178.81 (11)	C5—C6—C7—C8	−177.76 (12)
C1^i^—C3—C4—C5	172.08 (12)	C7—C6—C11—C10	−0.89 (18)
C2—C3—C4—C5	−9.4 (2)	C7—C6—C5—C4	170.84 (13)
C3^i^—C1—C2—C3	0.34 (19)	C12—O1—C1—C2	4.15 (17)
C3—C4—C5—C6	−179.01 (12)	C12—O1—C1—C3^i^	−176.23 (11)
C10—C9—C8—C7	0.7 (2)		

Symmetry code: (i) −*x*+2, −*y*+1, −*z*+1.
